# Secure Method for Biometric-Based Recognition with Integrated Cryptographic Functions

**DOI:** 10.1155/2013/623815

**Published:** 2013-05-15

**Authors:** Shin-Yan Chiou

**Affiliations:** Department of Electrical Engineering, Chang Gung University, 259 Wen-Hwa 1st Road, Kwei-Shan, Taoyuan 333, Taiwan

## Abstract

Biometric systems refer to biometric technologies which can be used to achieve authentication. Unlike cryptography-based technologies, the ratio for certification in biometric systems needs not to achieve 100% accuracy. However, biometric data can only be directly compared through proximal access to the scanning device and cannot be combined with cryptographic techniques. Moreover, repeated use, improper storage, or transmission leaks may compromise security. Prior studies have attempted to combine cryptography and biometrics, but these methods require the synchronization of internal systems and are vulnerable to power analysis attacks, fault-based cryptanalysis, and replay attacks. This paper presents a new secure cryptographic authentication method using biometric features. The proposed system combines the advantages of biometric identification and cryptographic techniques. By adding a subsystem to existing biometric recognition systems, we can simultaneously achieve the security of cryptographic technology and the error tolerance of biometric recognition. This method can be used for biometric data encryption, signatures, and other types of cryptographic computation. The method offers a high degree of security with protection against power analysis attacks, fault-based cryptanalysis, and replay attacks. Moreover, it can be used to improve the confidentiality of biological data storage and biodata identification processes. Remote biometric authentication can also be safely applied.

## 1. Introduction

Various aspects of everyday life are gradually being digitized as our life experiences and creative efforts are accumulated in personal computers, digital media devices, and mobile devices. People use passwords and other authentication methods to protect these collections of personal and potentially confidential information. Traditional confidentiality and authentication methods (e.g., personal passwords) are less than secure. In addition to requiring the user to remember a variety of passwords, which can result in user error, passwords can be stolen and pure password authentication is vulnerable to unauthorized breach. However, these problems can be resolved through the use of “physiological passwords” through unique personal biometric identification methods such as recognition of the user's face, fingerprints, personal signature, or iris, which are very difficult to either replicate or steal. Therefore, several biometrics-based remote user authentication schemes [1–9] have been designed. 

In general, however, traditional biometric identification methods only allow for direct comparison via a proximal end-user device and cannot be combined with cryptographic techniques. As long as biometric techniques allow for a degree of tolerance for error, the data are subject to disruption, rendering it impossible to accurately compare the scanned input with the original sample. In addition, registering the biometric feature values of the original biometric data to the biometric device for certification may encounter the following threats.

1. Hackers could crack the registered biometric feature data stored on the biometric device.
2. During matching, biometric data has a tolerance for error, making it impossible to use more secure means of encryption such as hash functions to protect biometric data, thus potentially allowing attackers to monitor private biometric data during the process of remote authentication.

In 2002, Lee et al., [1] proposed a type of remote authentication method based on fingerprints and smart cards. However, this method required precise system time synchronization. Later, in 2003, Kim et al., [10] proposed an ID-based authentication system integrating smart cards, passwords, and fingerprints. This system, however, was vulnerable to power analysis attacks [11] or fault-based cryptanalysis [1, 12]. At the same time, Scott [13] showed how this system was vulnerable to replay attacks. 

In 2010, Li and Hwang [7] proposed a biometrics-based remote user authentication scheme using smart cards. However, in 2011, Das [8] pointed out that their scheme is insecure due to the security drawbacks in password change phase and in verification of biometrics and proposed another improved scheme which provides mutual authentication and is secure against attacks of server masquerading, parallel session, and the stolen password. However, in 2012, An [9] showed that Das's scheme [8] does not provide mutual authentication and is vulnerable to various attacks and proposed enhanced scheme to solve their security problems.

This paper presents a new secure authentication method applying cryptographic techniques to biometric feature. The proposed method combines the advantages of biometric identification and cryptography. By adding a subsystem to existing biometric systems, the proposed approach achieves the high security of cryptographic techniques and the tolerance for error of biometric recognition.

For example, this method can be combined with dual-factor biometric and cryptographic identification to achieve security. This not only simultaneously provides biometric and cryptography authentication but also during the authentication process protects the biometric data through cryptographic encryption (e.g., hash). This method provides a high degree of security and is resistant to power analysis attacks, fault-based cryptanalysis, and replay attacks. Because the proposed method can be combined with cryptographic techniques, the biometric authentication can also apply cryptography techniques to ensure secure remote biometric matching.

Once the method has been integrated, if an attacker seeks to force access to obtain the database's presaved biometric feature data, the attacker can only get access to the hashed or encrypted confidential information. By applying this method, biometrics can be combined with a cryptographic system thus enhancing the secure storage and use of biological feature data and effectively preventing malicious programs or attackers from stealing the biometric values or posing as legitimate users. 

The proposed method combines biometrics matching to achieve cryptographic functions, such as encryption, authentication, identification, signature, hash, and key generation, which can be used in banks to replace IC cards, seals, and other means of dual identification, thus ensuring privacy, integrity, nonrepudiation, and so forth. These technologies can be implemented through hardware or software applications and combine biometric systems in current use. Thus, the contributions of the proposed method are as follows.

1. Simultaneously achieve the functions of cryptography technology and biometric recognition.
2. Cryptography operations for biometric data encryption, signatures, and so forth.
3. Error tolerance in biometric data matching.
4. Easily integrated into existing biometric systems.
5. Strengthens the confidentiality of biometric data storage. Even if an attacker accesses the registered biometric data stored in the biometric device, he will be unable to decrypt the biometric data or impersonate an authorized user.
6. Strengthens the confidentiality of biological information in the recognition process. Even if an attacker intercepts data during the biometric matching process, he will be unable to decrypt the biometric data or impersonate an authorized user.
7. Reduces vulnerability to power analysis attacks, fault-based cryptanalysis, and replay attacks.
8. Can be safely used to maintain confidentiality in remote biometric authentication. Even if an attacker eavesdrops during the remote authentication process, he will be unable to access biometric data or impersonate an authorized user.
9. Combines biometric recognition with cryptography technology but does not influence the error accept rate (EAR) or error reject rate (ERR) of the original biometric recognition.

### 1.1. Difference between Biometric Recognition and Cryptography Authentication

Biometric systems refer to the use of biometric recognition technology to authenticate a person's identity through his or her unique biological characteristics (e.g., fingerprints, palm prints, iris, personal signature) in lieu of a password. This approach can thus authenticate the user's identity without requiring the user to remember multiple passwords. This authentication method usually first obtains a threshold range to discriminate between acceptable and unacceptable inputs. However, repeated use, improper storage, or transmission leaks may compromise security.

The difference with cryptographic technology is that these authentication ratios do not need to achieve 100% accuracy. That is, a certain degree of error in data matching is tolerated. (Biometric and cryptography authentication methods are compared in Table 1.)

## 2. Related Work

### 2.1. Traditional Biometric Methods

**Figure 1:**
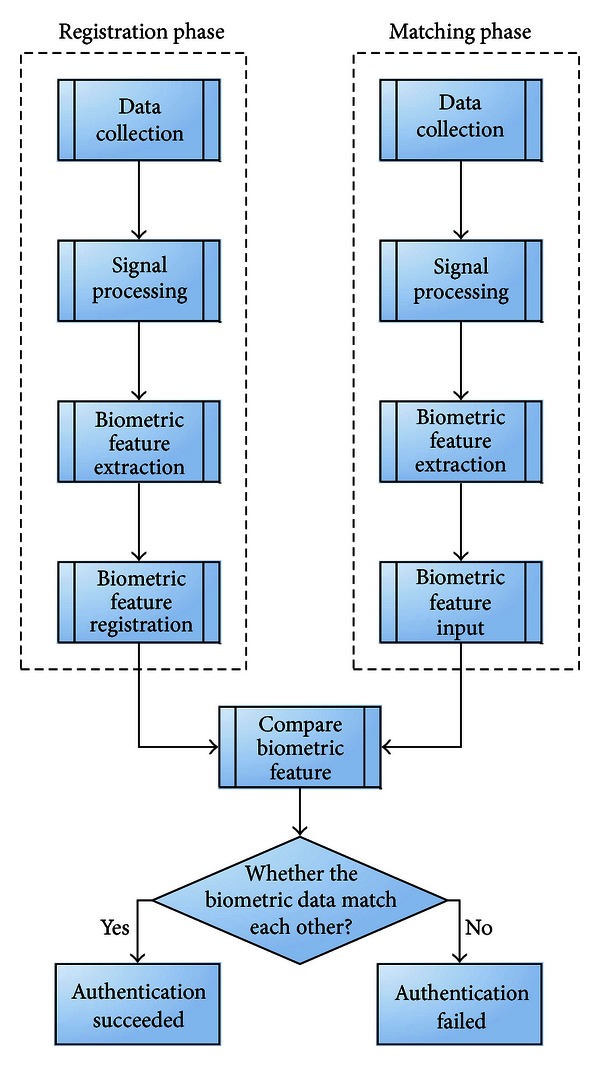
The processing of a conventional biometric method.

As shown in Figure 1, the processes of traditional biometric methods include the following subsystems: (1) data collection, (2) signal processing, (3) biometric feature extraction, (4) biometric feature registration/biometric feature input, and (5) matching and decision (i.e., comparing biometric features to determine whether they match). Generally speaking, one needs to first register/store biometric feature data (in the registration phase) for matching. Once this is completed, the biometric device allows the user to input his or her biometric feature data (in the matching phase) for comparison of the biometric features against those in the registration phase (in the compare biometric feature function) to determine if they match. If the biometrics of the prestored biometric features in the registration phase and those in the matching phase inputted by the user are found to match, then the device outputs a recognition result of “Authentication Successful.” Otherwise, the biometric device outputs a recognition result of “Authentication Failed.” Generally speaking, the steps in the registration phase and in the matching phase are processed similarly. For example, the matching phase is divided into the following steps: data collection, signal processing, biometric feature extraction, and biometric feature input. In terms of biometric feature matching, for the matching of the biometric feature registration data and the biometric feature input data, biometric authentication usually determines acceptability based on a threshold value.

Biometrics differs from cryptographic techniques in that, for biometric authentication, the ratio of credential matching does not need to be 100%; that is, the match between the two data sets can tolerate a certain degree of error. For example, suppose a registered biometric feature of 35 and a threshold value of 5, if the inputted biometric feature is within the range of 30 to 40, it is considered a biometric match with the registered biometric feature. However, if the biometric data is below 30 or exceeds 40, it is determined to be inconsistent with the registered feature values. In cryptographic authentication, if the registered password is 35 and the input value is 37, the input is considered to be inconsistent with the registered password, and the only allowable match would be an input value of 35.

As shown in Figure 1, the biometric processing device integrated with cryptographic technology consists of the following five parts: (1) data collection subsystem, (2) signal processing subsystem, (3) biometric feature extraction subsystem, (4) biometric feature registration/input subsystem, and (5) matching and decision subsystem.

1. Data Collection Subsystem
2. The data collection subsystem collects the registered biometrics (e.g., fingerprints, facial image, iris image) for matching with the input biometric. The subsystem generally uses a biometric sensor to read one or more aspects of the subject's biometric data.
3. Signal Processing Subsystem
4. The signal processing subsystem reads the biometrics and processes them through actions such as Gaussian smoothing, histogram equalization, normalization, binarization, opening, thinning, thinning repair, and feature point retrieval.
5. Biometric Feature Extraction Subsystem
6. A given biometric consists of many types of features such as the terminal and bifurcation points of fingerprint minutiae. General algorithms are used to retrieve the terminal and bifurcation points for feature matching. The biometric feature extraction subsystem is used to match the feature points or feature values of the retrieved biometric features.
7. Biometric Feature Registration/Input Subsystem
8. The biometric feature registration subsystem stores the processed biometric features for future identification. The biometric feature input subsystem stores the inputted and processed biometric features for comparison in next step.
9. Matching and Decision Subsystem
10. The matching and decision subsystem matches the inputted and processed biometric features with the registered biometric features stored in the database. If the match meets the required conditions, the match is validated.

### 2.2. Fingerprint Recognition

Biometric identification can be accomplished through the recognition of various characteristics including fingerprints and palm prints. Fingerprint minutiae are composed of the fine geometric features created by fingerprint ridges. Early on, Galton proposed identifying fingerprints based on four types of features: the beginnings and ends of ridges, forks, islands, and enclosures. However, Hrechak and Mchugh later proposed the use of eight minutiae: terminals, bifurcation, short ridges, crossovers, spurs, dots, islands, and bridges (see Table 2).

**Figure 2:**
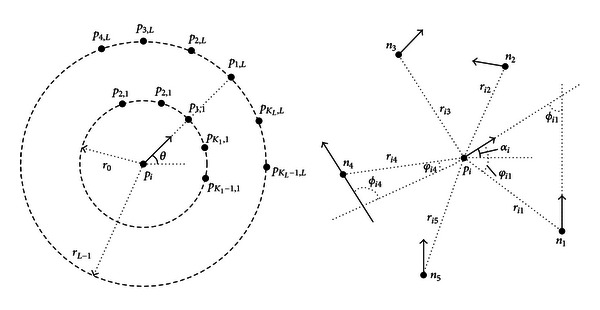
Structural matching methods.

Fingerprint recognition uses minutiae-matching algorithms such as the alignment-based matching algorithm [14], the Gabor filter-based approach [15], and the structural matching algorithm [16–19]. Among these, the structural matching algorithm (see Figure 2) is roughly divided into two stages. The first stage uses local feature matching to identify a central feature point with a positioning effect, while the second stage compares all the features at this central point and calculates a matching score.

### 2.3. Biometric-Based Cryptographic Key Generation

**Figure 3:**
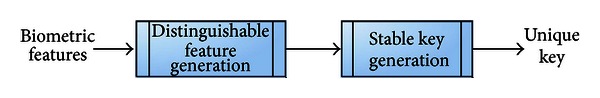
Structure of cryptography key generation based on biometric features.

**Figure 4:**
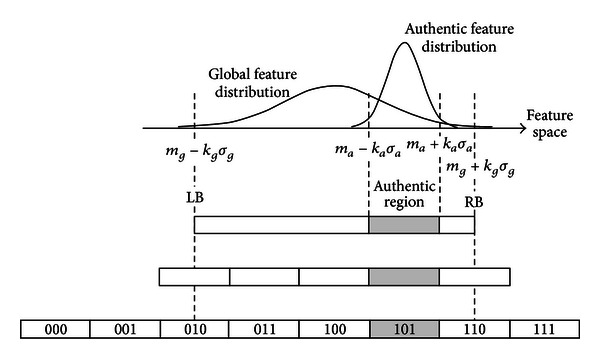
Example of cryptography key generation mechanism.

Chang et al. [20] proposed using a collected number of biometrics as a training sample to achieve “biometric-based cryptographic key generation.” As shown in Figures 3 and 4, this method uses multiple biometrics (including those for legitimate users) to find a conversion set through a mechanism which identifies highly distinguishing features. This allows each one-dimensional feature of the postbiometric conversion to effectively distinguish between legitimate and illegitimate users. The average features of legitimate users are then used to authenticate the identity of the legitimate user as a mechanism for generating multibyte passwords. (This group conversion must be stored in the biometric database.) However, this approach must be applied to the biometric data of multiple users to achieve differentiation. Also, because the error value calculation is determined based on the mean and variance of each biometric, therefore each user must provide multiple biometric samples to generate the associated means and variances.

### 2.4. Fuzzy Extractors

Dodis et al. [21] proposed a cryptographic key generation mechanism called fuzzy extractors. This system uses biometric values and self-selected authentication values as input data. During recognition, it uses a cryptographic key and self-selected authentication values to recognize biometric values within a set error range. Furthermore, this system can use cryptographic keys and input biometric values (within a predetermined error range) to restore the original biometric values.

**Figure 5:**
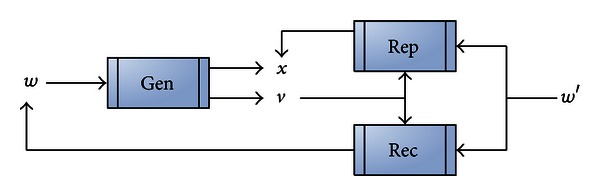
Fuzzy exactor.

As shown in Figure 5, this method first selects an authentication value *x* and then uses the Gen function, with *x* and the registered biometric value *w* to generate a key *v* as follows:

(1) $$\begin{array}{c} \text{Gen}:v=w⊕C\left(x\right), \end{array}$$

where *C*(·) is the encoding function of a type of error correction code (e.g., Hamming code).

Next, within an error range *t*, using the Rep function causes *v* and *x* to recognize the inputted biometric value *w*′ (where distance (*w*, *w*′) ≤ *t*). The Rep function is as follows:

(2) $$\begin{array}{c} \text{Rep}:D\left(w^{\prime }⊕v\right)=x, \end{array}$$

where *D*(·) is a type of error correction decoding function.

In case the original biometric value *w* is lost, *w* can be restored through inputting biometric value *w*′ of the cryptographic key *v* and the error range *t* through the Rec function. The Rec function is as follows:

(3) $$\begin{array}{c} \text{Rec}:\text{Rec}\left(w^{\prime },v\right)=v⊕C\left(D\left(w^{\prime }⊕v\right)\right)=w_{\circ }. \end{array}$$

However, this method cannot be integrated into current biometric systems. Moreover, this method's operating system not only requires the use of key *v* and authentication value *x* to perform authentication (and thus requires the storage of key *v*), but this comparison method is also vulnerable to leaking biometric value *w* (through the use of biometric value *w*′ and key *v*).

### 2.5. Application to Combine Iris Recognition and Cryptography

**Figure 6:**
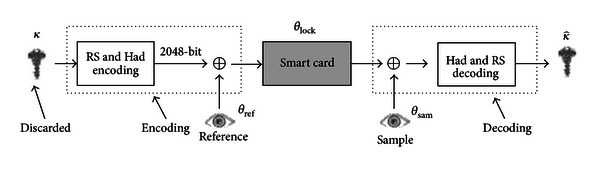
Iris recognition combining cryptography.

Hao et al., [22] proposed an application combining iris recognition and cryptography (see Figure 6). The concept for this method is similar to that of the fuzzy extractor in that they both use an error control code to accept biometric values within a range of errors.

This system first uses a cryptographic key *κ* and the iris biometric value *θ*_ref_ to obtain the authentication value *θ*_lock_ and stores *θ*_lock_ and the key's hash value *h*(*κ*) in the IC card, based on the following relationship:

(4) $$\begin{array}{c} \theta_{\text{lock}}=\theta_{\text{ps}}⊕\theta_{\text{ref}}, \end{array}$$

where *θ*_ps_ is the value for the key *κ* via RS and Hadamark coding.

During recognition, the XOR value of *θ*_lock_ and the inputted iris biometric value *θ*_sam_ can be decoded as *κ*′ through RS and Hadamark decoding to determine if *h*(*κ*′) is equal to *h*(*κ*). If the difference between the inputted iris biometric value *θ*_sam_ and the original iris biometric value *θ*_ref_ is less than or equal to a tolerable error range of the error control code, thus the input will be decoded as the original *κ* value and considered correct.

However, this method is only suitable for iris matching and cannot be directly combined with existing systems. The RS code is used as a means to calculate network transmission errors for each byte, which differs from error calculation methods in other biometric environments.

## 3. Proposed Scheme

This paper presents a secure cryptography-integrated biometric recognition method with cryptographic functions. This method is able to integrate biometric matching with cryptographic technology to achieve dual-factor authentication. This integrated technology can also be combined with more advanced cryptographic techniques to produce more secure and diverse applications. The proposed method is divided into two parts for description purposes. The first part is basic process of improved biometric security (IBS), while the second part is advanced process of integrated cryptographic technology (ICT).

The IBS process is divided into two phases: the registration phase and the matching phase. The registration phase first provides a set of biometric data. Based on a threshold value *t*, we define several numerical ranges, each of which has a quantization value. If the biometric data fall within one of these numerical ranges, then the quantized value for that numerical range is used as a quantized feature data to replace the biometric feature data. Next, one-way function operations are used to convert the quantized feature data to hashed feature data (*H*_*F*_). Then, the difference between the quantized feature data and the biometric data is calculated to obtain an adjustment value (*V*_AD_). Finally, this adjustment value *V*_AD_ is stored with the hashed feature data *H*_*F*_.

Matching phase and registration phase are largely similar. First we provide a registered hashed feature data *H*_*F*_ and adjustment value *V*_AD_, and the biometric data is then captured. The biometric data is adjusted based on this adjustment value *V*_AD_. Next, (similarly) based on the threshold value *t*, multiple numerical ranges are defined, each of which is a quantized value. If the adjusted biometric data fall within one of the numerical ranges, then the quantized value of this value range is taken as the quantized feature to replace the adjusted biometric data. This is followed by one-way function operations to convert the quantized feature into hashed feature data *H*_*F*_′. Finally, the registered hashed data *H*_*F*_ is compared with the hashed feature data *H*_*F*_′.

In the ICT process, the biometric data must first go through IBS process before it can be used in this process. This process integrates the cryptography technology for signature application using the biometric data, which is composed of the “registration” and “signature and verification” stages. The application provides biometric-based cryptographic fields for the signatory and the verifier. 

Before describing the processes of IBS and ICT, we define the notations used in our proposed protocol in Table 3. 

### 3.1. Process of Improved Biometric Security (IBS)

To improve the security of storage of biometric feature data, biometric feature values must first be processed before being integrated with cryptography technology. This method uses numerical quantization and quantization adjustment processes to ensure that all acceptable values within the threshold are quantified to the same value without compromising security. This quality can use hash or encryption functions to prevent the theft or leakage of the registered data prestored in the database. During matching, the values must be exactly correct in order to pass, thus improving the comparison rate of hardware or software. Because some biometric values are quantized to a correct value without error, these values not only can use hash or encryption functions for protection but can also be further applied through other cryptographic techniques or other numerical derivations such as signatures, key generation, and key exchange. 

**Figure 7:**
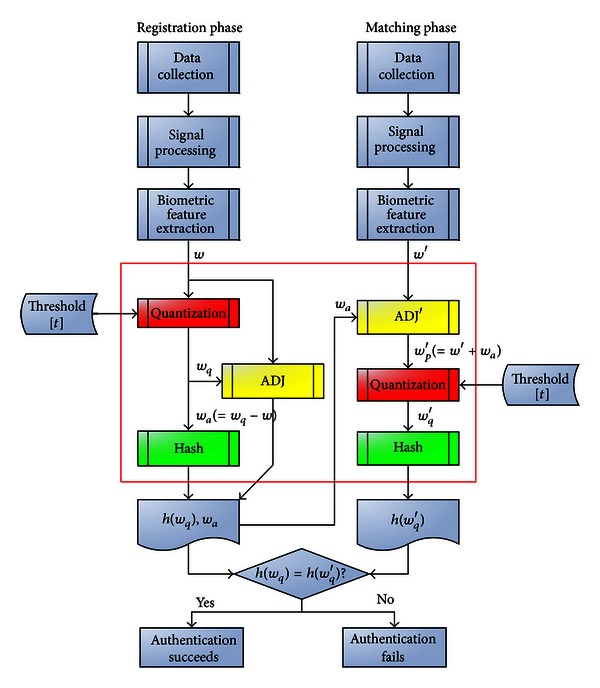
Schematic diagram of the processing of the proposed method.

Figure 7 shows a schematic diagram of the biometric processing methods of the proposed cryptography-integrated technology. The processed values can be directly applied to biometric recognition. This processing mode (shown in Figure 7) can be divided into eight parts as follows: (1) data collection subsystem, (2) signal processing subsystem, (3) biometric feature extraction subsystem, (4) numerical quantization subsystem, (5) adjustment subsystem, (6) hash subsystem, (7) biometric feature registration/input subsystem, and (8) matching and decision subsystem, where (1) the data collection subsystem, (2) the signal processing subsystem, and (3) the biometric feature extraction subsystem are the same as those mentioned in Section 2.1. Thus, below, we limit our explanation to subsystems (4)–(8).

(4) Numerical Quantization Subsystem
 The numerical quantization subsystem performs value quantization on the processed signal (as *w*_*q*_ and *w*_*q*_′). These quantized values can then be used with cryptographic techniques. Assume that the signal comparison allows for an error range of plus or minus *t* and a sampling value range between (0, *L*). Then the interval of the quantitative mode is *p*, the signal value is quantized as 0, *p*, 2*p*,…, *np*, where *p* = 2*t*, *n* = ⌊*L*/*p*⌋ (where ⌊·⌋ is a floor function). If a signal value *w* between (0, *L*) satisfies (*kp* − *p*/2) ≤ *w* < (*kp* + *p*/2), then this signal value *w* should be quantized as *w*_*q*_ = *kp*. For example, for some signal value (28, 37, 19, 62, 54) and *t* = 5 (i.e., *p* = 10), the signal value is quantized as (30, 40, 20, 60, 50). (Generally speaking, if a biometric value allows an error range of ±*t*, then*p* = 2*t* can be used to obtain the quantization interval.) If the quantized range defined by the threshold is used for quantization, then the ERR and EAR obtained using this method will have no impact.
(5) Adjustment Subsystem
 The adjustment subsystem records the fine-tuned value *w*_*a*_ from the quantizing process. This fine-tuned value can be quantized to restore the reduced recognition rate to the original recognition rate without compromising security. The recommended calculation method for the fine-tuned value is *w*_*a*_ = *w*_*q*_ − *w*. For example, given a signal value *w* = (28,37,19,62,54) and *p* = 10, the signal value is quantized as *w*_*q*_ = (30, 40, 20, 60, 50), then the adjustment value *w*_*a*_ is (2, 3, 1, −2, −4). Given an inputted value *w*′ = (24, 33, 21, 66, 58), *p* = 10, and the adjustment value *w*_*a*_ = (2, 3, 1, −2, −4), then the adjusted value *w*_*p*_′ = (26, 36, 22, 64, 54) which is quantized as *w*_*q*_′ = (30, 40, 20, 60, 50).
 Using the numerical quantization and adjustment process guarantees that all accepted values remain within the threshold value and are quantized at the same level of quality without compromising security. (Given an acceptable error range of plus or minus *t*, correctly guessing a value between a sampling value (0, *L*) has a probability of approximately 2*t*/*L*; following quantization, correctly guessing the quantized value between a sampling value of (0, *L*) has a probability of approximately 1/*n*, where *n* = ⌊*L*/*p*⌋ = ⌊*L*/2*t*⌋. The probability of correctly guessing the un-quantized value is identical to that of the quantized value; therefore, the quantized action does not compromise security.)
(6) Hash Subsystem
 The value *w*_*q*_ produced by the hash function is *h*(*w*_*q*_). Using the hash function can maintain biometric confidentiality and prevent leaking or theft of the presaved registered feature values stored in the database. Because a hacker would only be able to manage the registered feature data stored in the biometric device, he would be unable to obtain the original biometric value. During comparison, the values must be exactly correct in order to pass, thus improving the hardware or software comparison rate. Other functions (e.g., encryption functions) can be used to substitute for this hash function.
(7) Biometric Feature Registration/Input Subsystem
 Applied to the proposed method, the stored values for registration are *h*(*w*_*q*_) and *w*_*a*_. This function is similar to the one previously described in Section 2.1.
(8) Matching and Decision Subsystem
 Applied to the proposed method, this system's comparison mode determines whether *h*(*w*_*q*_) and *h*(*w*_*q*_′) are the same. This function is similar to the one previously described in Section 2.1.

Figure 1 shows the processing of a conventional biometric method, while Figure 7 demonstrates schematic diagram of the processing of the proposed method. As shown in Figure 1, a threshold value and a biometric matching method decide the EAR and ERR. We combine threshold and quantization (as shown in Figure 7) to quantify registered and input biodata within threshold to the same value and use biometric matching methods to compare data after hashing these values. Therefore, the hashed values can be applied to cryptography technology, and the combination of biometric recognition and cryptography technology does not influence the EAR or ERR of the original biometric recognition.

### 3.2. Process of Integrated Cryptographic Technology (ICT)

Once the complete quantified features have been hashed (in biometric feature registration subsystem), dual authentication can be achieved through the integration of cryptographic techniques. This method can be separated into a “registration” phase and a “signature and authentication” phase as follows.

#### 3.2.1. Registration Phase

**Figure 8:**
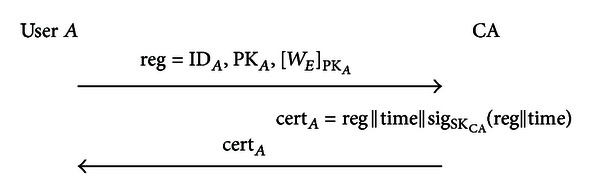
Registration phase.

As seen in Figure 8, user *A* first personally registers with CA and transmits message reg = ID_*A*_, PK_*A*_, [*W*_*E*_]_PK_*A*__ to CA, where ID_*A*_ is the ID of user *A*, PK_*A*_ is user *A*'s public key, *W*_*E*_ is the registered and internally stored biodata to be recognized, and [*W*_*E*_]_PK_*A*__ represents the encrypted signal *W*_*E*_ using the user's public key PK_*A*_. Next, CA's certificate cert_*A*_ = reg||time||sig_SK_CA__(reg||time) is transmitted to user *A*, where sig_SK_CA__(*M*) represents the signature of signal *M* using CA's private key SK_CA_, and time represents the certificate's validity period.

#### 3.2.2. Signature and Verification Phase

Generally speaking, a single type of biometric comparison may have more than one matching stage (e.g., structural comparison has a dual-stage comparison). Assume that this biometric has two stages, the stage *j* matching requires data *W*_*E*_^(*j*)^ and *W*_*I*_^(*j*)^, where *W*_*E*_ is the internal registered data and *W*_*I*_ is the input biometric data used for matching the internal data.

**Figure 9:**
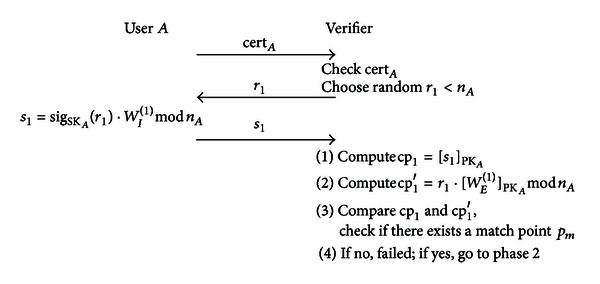
Comparison process of first stage.

**Figure 10:**
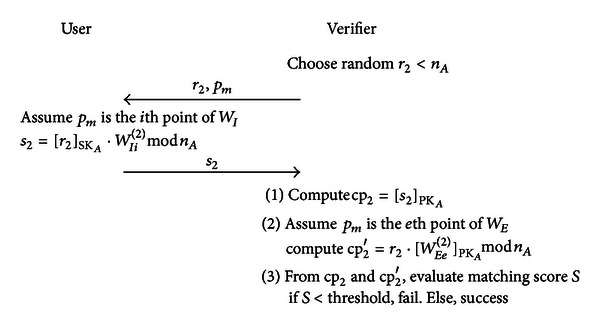
Comparison process of second stage.

1. First stage comparison
2. As seen in Figure 9, user *A* first sends cert_*A*_ to the verifier. Then the verifier confirms the accuracy of cert_*A*_ and selects a random number *r*_1_ to send to user *A*. Next, *A* calculates *s*_1_ = sig_SK_*A*__(*r*_1_) · *W*_*I*_^(1)^mod⁡*n*_*A*_ and sends this to the verifier, where *n*_*A*_ is the product of two large prime numbers used as one of *A*'s public keys. Finally, the verifier separately calculates cp_1_ = [*s*_1_]_PK_*A*__ and cp_1_′ = *r*_1_ · [*W*_*W*_^(1)^]_PK_*A*__mod⁡*n*_*A*_, and compares cp_1_ and cp_1_′, to determine whether there exists a match point *p*_*m*_. If there exists a match point, go to the second stage; otherwise terminate this stage.
3. Second stage comparison
4. As seen in Figure 10, the verifier first selects a random number *r*_2_, which it sends with *p*_*m*_ to *A*. Assume that *p*_*m*_ is the *i*th point in *W*_*I*_^(1)^, then *A* calculates *s*_2_ = [*r*_2_]_SK_*A*__ · *W*_*Ii*_^(2)^mod⁡*n*_*A*_, and sends *s*_2_ to the verifier, where *W*_*Ii*_^(2)^ is related data value of the *i*th point of *W*_*I*_^(2)^ for *W*_*I*_ in the second stage matching.

Next, the verifier calculates cp_2_ = [*s*_2_]_PK_*A*__. Assume *p*_*m*_ is the *e*th point in *W*_*E*_, then the verifier calculates cp_2_′ = *r*_2_ · [*W*_*Ee*_^(2)^]_PK_*A*__mod⁡*n*_*A*_ and compares cp_2_ and cp_2_′ to calculate a matching score *S*. If *S* is smaller than the threshold, then verification fails; otherwise, verification is successful.

If a biometric matching method has only one stage, then the first stage matching allows for the calculation of a matching score. If a biometric matching method has three, four, or more stages, then, after the second stage, the verifier continues to select and send random numbers *r*_3_, *r*_4_, and so forth to the user. The user then similarly calculates and sends *s*_3_, *s*_4_, and so forth to the verifier to obtain a final matching score.

## 4. Analysis of Proposed Scheme

### 4.1. Security Analysis

We analyze the security of our protocols according to the requirements of contributions expressed in Section 1 as follows.

#### 4.1.1. Strengthens the Confidentiality of Biometric Data Storage

Since only *h*(*w*_*q*_) and *w*_*a*_ are registered and stored, even if an attacker accesses the registered biometric data stored in the biometric device, he will be unable to decrypt the biometric data or impersonate an authorized user.

#### 4.1.2. Strengthens the Confidentiality of Biological Information in the Recognition Process

Because only *w*_*a*_ is transmitted and *h*(*w*_*q*_) is compared during the biometric matching process, even if an attacker intercepts data during the process, he will be unable to decrypt the biometric data or impersonate an authorized user.

#### 4.1.3. Reduces Vulnerability to Power Analysis Attacks, Fault-Based Cryptanalysis, and Replay Attacks

Since only *h*(*w*_*q*_) and *w*_*a*_ are registered and stored, an attacker will be unable to use power analysis attacks or fault-based cryptanalysis to break the system. Moreover, because different random numbers *r*_*i*_ are used in each matching process (as seen in Figures 9 and 10), even if an attacker eavesdrops during the process, he will be unable to use these data to access biometric data or impersonate an authorized user. Therefore, this system is replay-attack resistant.

#### 4.1.4. Can Be Safely Used to Maintain Confidentiality in Remote Biometric Authentication

As only *w*_*a*_ is transmitted and different random numbers *r*_*i*_ are used to protect biometric data during remote biometric authentication process (as shown in Figures 9 and 10), even if an attacker eavesdrops during the process, he will be unable to access biometric data or impersonate an authorized user.

### 4.2. Comparison

According to the nine contributions expressed in Section 1, we compare our protocol with the protocols of biometric-based cryptographic key generation (BCKG) [20], fuzzy extractors (FZ) [21], and application to combine iris recognition and cryptography (ACIRC) [22]. The results are summarized in Table 4, where Tech. and (1)–(9), respectively, denote technique and the nine contributions described in Section 1. As seen in Table 4, all schemes offer the error tolerance in biometric data matching (as shown in item (3)) because the main usage of these schemes are in biometric matching. As seen in items (2), (4), (8), and (9), only the proposed scheme provides these functions since our scheme is used to integrate into existing biometric systems with confidentiality and cryptography technologies.

## 5. Applications of the Proposed Method in Structural Comparison

Some methods for biometric identification are suitable for use in the proposed method (e.g., minutiae matching algorithms such as structural matching algorithm [23, 24], the improved structural matching algorithm [25, 26], and the onion layer algorithm [27–29]).

If the proposed method is used in the structural matching algorithm, the first stage matching content is hashed before matching, and the first stage matching results obtain the optimal core position, which is then used in the second stage matching. Similarly, the second stage matching content can also be hashed before matching. If the quantitative range set by the threshold is used for quantization, then the ERR and EAR will not change with the application of this method. As an example, the structural matching algorithm is applied to the proposed method.

The structural matching algorithm is divided into two stages. The first stage matches local features to identify a core point with the positioning effect. The second stage uses this core point to conduct overall feature matching and obtain a matching score. 

**Figure 11:**
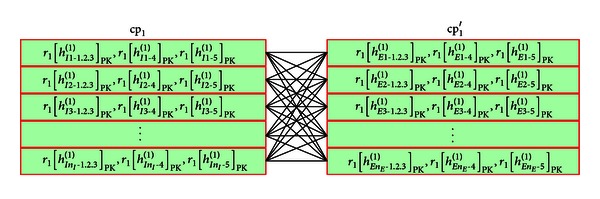
First stage matching content.

For example, assume that the number of feature points of the input and registered fingerprint are *n*_*I*_ and *n*_*E*_, respectively, and assume that first stage takes five matching data. Then *W*_*I*_^(1)^ = *W*_*I*1_^(1)^ | |*W*_*I*2_^(1)^ | |⋯||*W*_*In*_*I*__^(1)^ and *W*_*E*_^(1)^ = *W*_*E*1_^(1)^ | |*W*_*E*2_^(1)^ | |⋯||*W*_*En*_*E*__^(1)^, where *W*_*Ij*_^(1)^ = *w*_*Ij*1_^(1)^ | |*w*_*Ij*2_^(1)^ | |*w*_*Ij*3_^(1)^ | |*w*_*Ij*4_^(1)^ | |*w*_*Ij*5_^(1)^ and *W*_*Ej*_^(1)^ = *w*_*Ej*1_^(1)^ | |*w*_*Ej*2_^(1)^ | |*w*_*Ej*3_^(1)^ | |*w*_*Ej*4_^(1)^ | |*w*_*Ej*5_^(1)^. Using the hash function we can let *h*_*Ej*-1.2.3_^(1)^ = hash⁡(*w*_*Ej*1_^(1*q*)^ | |*w*_*Ej*2_^(1*q*)^ | |*w*_*Ej*3_^(1*q*)^), *h*_*Ej*-4_^(1)^ = hash⁡(*w*_*Ej*4_^(1*q*)^), *h*_*Ej*-5_^(1)^ = hash(*w*_*Ej*5_^(1*q*)^) and *h*_*Ij*-1.2.3_^(1)^ = hash⁡(*w*_*Ij*1_^(1*q*)^ | |*w*_*Ij*2_^(1*q*)^ | |*w*_*Ij*3_^(1*q*)^), *h*_*Ij*-4_^(1)^ = hash⁡(*w*_*Ij*4_^(1*q*)^), *h*_*Ij*-5_^(1)^ = hash⁡(*w*_*Ij*5_^(1*q*)^), where *w* ^(1*q*)^ represents the quantized value of *w* ^(1)^. Then Figure 11 shows the matching of cp_1_ and cp_1_′. 

In the second stage matching, we can let *W*_*Ij*_^(2)^ = {hash⁡(*w*_*Ij*1_^(2*q*)^) | |hash⁡(*w*_*Ij*2_^(2*q*)^) | |⋯||hash⁡(*w*_*Ijn*_*I*__^(2*q*)^) − hash⁡(*w*_*Ijj*_^(2*q*)^)}, *W*_*Ej*_^(2)^ = {hash⁡(*w*_*Ej*1_^(2*q*)^) | |hash⁡(*w*_*Ej*2_^(2*q*)^) | |⋯||hash⁡(*w*_*Ejn*_*E*__^(2*q*)^) − hash⁡(*w*_*Ejj*_^(2*q*)^)}, where *w*_*Ijl*_^(2)^ and *w*_*Ejl*_^(2)^ are the relationship values between the core point (the *j*th point) and its neighboring feature point (the *l*th point) (e.g., type, distance, relationship angle, etc.) for the input fingerprint and the registered fingerprint, respectively, in second stage matching, and *w*_*x*_^(2*q*)^ represents the quantized value of *w*_*x*_^(2)^.

## 6. Conclusions

This paper proposes a new biometric authentication method with the security of cryptographic technology, simultaneously achieving the functions of cryptographic technology and biometric recognition. This method is very simple to implement through the addition of a subsystem to existing biometric systems. The proposed method offers increased security, with resistance to power analysis attacks, fault-based cryptanalysis, and replay attacks. This method can also strengthen the confidentiality of stored biometric data and recognition processes and also offers secure remote biometric identity authentication. Fingerprint structural matching is presented as an application example for reference of a technical implementation. The proposed concept can be applied to any combination of biometrics and cryptographic techniques to securely exploit the advantages of both technologies.

## Figures and Tables

**Table 1:** Comparison between biometrics recognition and cryptography authentication.

	Cryptography authentication	Biometrics recognition
Authentication method	Digital	Analog
Authentication rule	Without error tolerance	With error tolerance
Data processing	Data is disordered	Data is processed but not disordered
Adoption of cryptography technique	Data can be encrypted and signed	Data cannot be encrypted or signed

**Table 2:** Eight types of fingerprint minutiae.

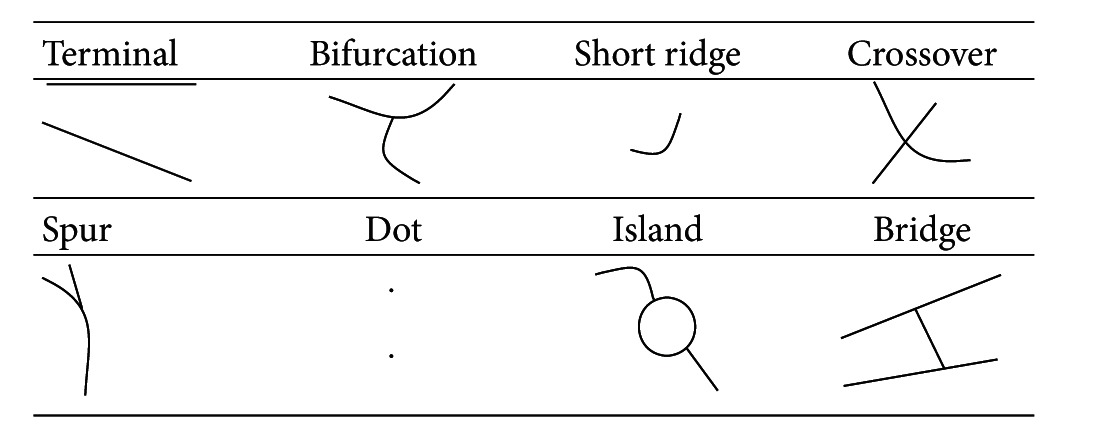

**Table 3:** Notations.

Notations	Meaning
*t*	Threshold value
*p*	The interval of the quantitative mode
*w*/*w*′	Biometric feature extraction data
*w*_*q*_/*w*_*q*_′	Data after value quantization
*w*_*a*_	Fine-tuned values
ID_*A*_	The ID of user *A *
PK_*A*_	The public key of user *A *
*W*_*E*_	Internal registered biodata to be recognized
*W*_*I*_	Input biodata for matching the internal biodata
*W*_*E*_^(*j*)^/*W*_*I*_^(*j*)^	*W*_*E*_/*W*_*I*_ in the stage *j *
*W*_*Ei*_^(*j*)^/*W*_*Ii*_^(*j*)^	Related data value of the *i*th point of *W*_*E*_^(*j*)^/*W*_*I*_^(*j*)^
cert_*A*_	Certificate of user *A *
time	Validity period of certificate
*n*_*A*_	Product of two large primes as *A*'s parameters
*h*(·)	Cryptographic one-way hash function
⌊·⌋	Floor function
[·]_PK_	Encryption function using public key PK
sig_SK_(·)	Signature using private key SK

**Table 4:** Comparison of functions.

Tech.	BCKG	FZ	ACIRC	Proposed scheme
(1)	*√*			*√*
(2)				*√*
(3)	*√*	*√*	*√*	*√*
(4)				*√*
(5)	*√*		*√*	*√*
(6)	*√*		*√*	*√*
(7)	*√*		*√*	*√*
(8)				*√*
(9)				*√*
